# Data Handling in Industry 4.0: Interoperability Based on Distributed Ledger Technology

**DOI:** 10.3390/s20113046

**Published:** 2020-05-27

**Authors:** Shengjing Sun, Xiaochen Zheng, Javier Villalba-Díez, Joaquín Ordieres-Meré

**Affiliations:** 1 Escuela Técnica Superior de Ingenieros Industriales (ETSII), Universidad Politécnica de Madrid, José Gutiérrez Abascal 2, 28006 Madrid, Spain; shengjing.sun@alumnos.upm.es (S.S.); xiaochen.zheng@epfl.ch (X.Z.); javier.villalba-diez@hs-heilbronn.de (J.V.-D.); 2 ICT for Sustainable Manufacturing, SCI-STI-DK, École polytechnique fédérale de Lausanne (EPFL), 1015 Lausanne, Switzerland; 3 Fakultaet fuer Management und Vertrieb, Campus Schwäbisch-Hall, Hochschule Heilbronn, 74081 Heilbronn, Germany; 4 Escuela Técnica Superior de Ingenieros Informáticos (ETSIInf), Universidad Politécnica de Madrid, Calle de los Ciruelos, Boadilla del Monte, 28660 Madrid, Spain

**Keywords:** industry 4.0, reference architecture model, interoperability, digital twin, distributed ledger technology, GDPR, RAMI 4.0, LASFA

## Abstract

Information-intensive transformation is vital to realize the Industry 4.0 paradigm, where processes, systems, and people are in a connected environment. Current factories must combine different sources of knowledge with different technological layers. Taking into account data interconnection and information transparency, it is necessary to enhance the existing frameworks. This paper proposes an extension to an existing framework, which enables access to knowledge about the different data sources available, including data from operators. To develop the interoperability principle, a specific proposal to provide a (public and encrypted) data management solution to ensure information transparency is presented, which enables semantic data treatment and provides an appropriate context to allow data fusion. This proposal is designed also considering the Privacy by Design option. As a proof of application case, an implementation was carried out regarding the logistics of the delivery of industrial components in the construction sector, where different stakeholders may benefit from shared knowledge under the proposed architecture.

## 1. Introduction

The recent advances in Information Technology, Internet of Things (IoT) and Cyber-Physical Systems (CPS), among other fields, have enabled digitization and automation of production processes and led to the definition of the fourth industrial revolution, also known as Industry 4.0 (I4.0) [1,2,3]. In the manufacturing domain, the I4.0 vision has promoted smart manufacturing and smart factories concepts by augmenting all assets with sensor-based connectivity [4]. Intelligent sensors such as positioning tags, safety gloves [5], head-mounted displays (HMDs), and smart glasses [6] have been widely applied in industrial applications. These intelligent sensors generate a large volume of industrial data and it remains a challenging task to collect, store, analyze, and exploit this data in business, including in simulations, virtual reality, digital twins, and so on [7].

Human factors, such as fatigue indicators, have significant effects on product quality and factory productivity in manufacturing activities [8]. It is clear that increasing the integration of data (including wearable information) can help to increase understanding of the performance of a business [9]. Indeed, when there is interest in increasing the existing information, a higher level of data integration considering both sub-systems (process and operator) is vital to achieve an effective and efficient human cyber–physical symbiosis [10]. Therefore, pervasive data interconnection and data-driven dynamic decision-making are the key points of Industrial Internet of Things (IIoT) infrastructures, in order to deliver a significant increase in business performance.

To ensure all participants involved in I4.0 share a common perspective and understanding, the *Reference Architecture Model for Industry 4.0* (RAMI 4.0) [11] was conceived as a standard architecture to describe the fundamental aspects of I4.0. It helps to leverage the process of transitioning classical manufacturing systems to I4.0. However, this model, as well as some existing frameworks [12,13], insufficient focus on those wearable devices in the integration layer, especially those related to human bio-sensing dimensions; in such a way, the value extended from data fusion from wearable devices is weak, from both functional and business points of view. Such imperfections mainly linked to aspects of privacy, data ownership, data silos and a lack of interoperability.

**Figure 1:**
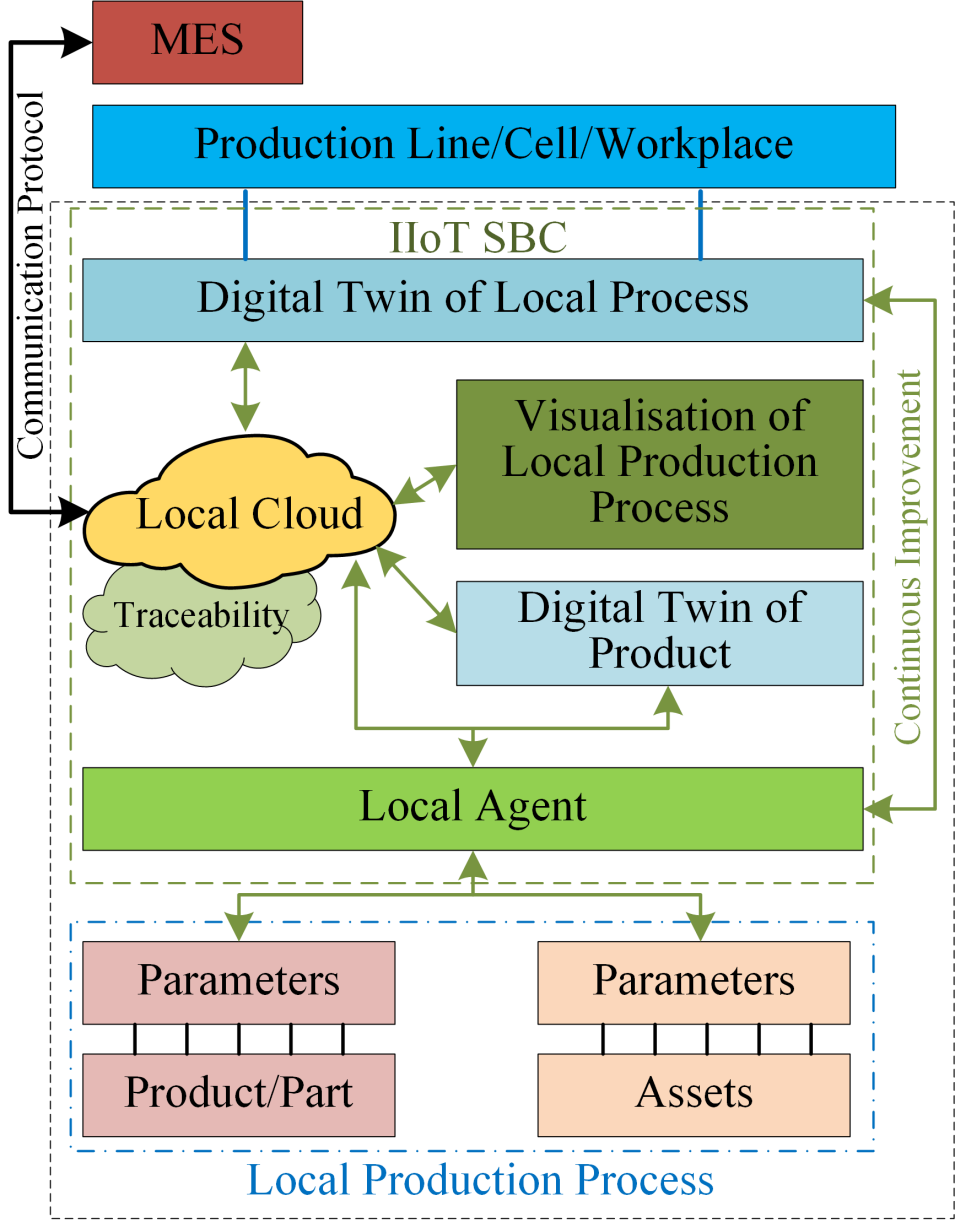
Lower level components of LASFA architecture model [14].

Recently, a new architecture model based on RAMI 4.0, named LASFA (LAsim Smart FActory), has been proposed [14]. It adopts the hierarchy of the layers from RAMI 4.0 and focuses on communication among the distributed systems in smart factories, which can help manufacturing companies to transform their manufacturing processes and systems towards the Industry 4.0 paradigm. Figure 1 presents an example of the lower level of such a local production system, as well as the data flows among different components. Compared with RAMI 4.0, the LASFA approach provides a simple configuration for smart factory-enabling agents and manufacturing units in different layers. Due to these advantages, the LASFA architecture model was selected for further development in this work.

Integration of data requires data fusion capabilities, where data fusion from different systems demands interoperability capacities of such systems, as this accounts for the capability of a system to work with other products or systems; either for implementation or access without significant restrictions [15,16].

Interoperability is related to heterogeneity in information theory. Therefore, classical taxonomies identified Semantic Interoperability, Structural Interoperability, Syntactic Interoperability, and System Interoperability [17]. The focus of this work is to enable the syntactic and structural dimensions of interoperability by fostering terminology transparency and context-sensitive information processing. This is due to the lack of common data standards (i.e., models, dictionaries, and conventions) which enable the interpretation of data across sub-systems inside an organization, hindering effective data re-usability under a standard format outside of the provided data environment. Therefore, addressing such a gap will help automatic information processes through CPS. The paradigm of cyber–physical equivalence (or digital twin) has emerged [18] to provide a comprehensive physical and functional description of a component, product, or system, in such a way that its information can be useful in any of its life-cycle phases [19].

When a value proposal requires the integration of different stakeholders with different Information Technology (IT) capabilities, such as customers or specific providers, granting access to a public database with immutable but encrypted data is a significant proposal. To this end, different cases can be figured out; on one hand, customers wish to have access not only to the product itself, but also to the production data of the product [20] for different reasons, such as knowledge of the environmental footprint, logistics, and so on.

On the other hand, providers of specific services (e.g., certification bodies) require certified production rates, environmental performance, or continuous improvement, which is another clear example of requiring access to such production-related data streams. Organizations such as regulatory agencies expect holistic and transparent information exchange with peer companies or factories. Furthermore, when asked, 88% of U.S. internet users and 87% of British internet users wish to control the data being collected through smart devices [21]. Encrypted but public databases enable data ownership of producers (especially those related to humans), which can ensure trust and business values resulting from data re-usability by other data consumers.

In modern interconnected societies, where different stakeholders aim to gather different views of different data flows and implement their workflows to make use of them, companies need to consider their value proposals in order to attract current and future stakeholders with relevant data, as well as being able to properly interpret them [22,23]. There is a need for clients, such as middleware platforms, end-users, services, and applications, which efficiently and effectively retrieve IIoT data resources, enabling the integrated and interoperable usage of their data streams.

Due to the different requirements that different stakeholders have in relationship to production-related data encoded into public databases, but also due to the different ways of processing such data, the convenience of integrating semantic meaning into the data itself has arisen. Thus, different stakeholders can easily integrate data processing into their own workflows, and the whole process becomes more resilient to changes in format, the creation of new attributes or entities, and so on. These aspects are linked to data transparency, understood (in the sense of [24,25,26]) as the degree of visibility and accessibility of information, which includes a component for providing an institutional infrastructure.

Of the secure and non-intensive technologies requiring third-party data access as previously described, the company itself may benefit from providing such standardized production process data flows; particularly when a lean management strategy is implemented. In the case of the usage of the (CPD)${}_{n}$A method [27,28,29], the expansion of Key Performance Indicator (KPI) at different levels through time can render traceability useful for the external certification of auditing processes. In addition, workflows can be activated based on their progress, as per the Value Stream Mapping method [30,31]. Therefore, the management dimension is also relevant for this proposal.

Under the described context, this paper aims to promote the principles of interoperability and data transparency in the I4.0 context by enabling the integration of data coming from different IIoT devices, including wearable devices, as well as enabling the integration from different data sources, with the aim of enhancing data fusion among processes from different stakeholders.

The contributions from this paper are the following:

- An enhanced framework providing flexibility to accommodate different data sources and digital twin, as well as an integrated mechanism to disseminate the desired content while limiting exposition of the related IT systems. This creates a common understanding of interoperability.
- Integration of ontologies with the data sets, enabling data fusion techniques and a higher degree of flexibility for data manipulation, enabling automation in machine learning applications.
- Integration of data dissemination in an encrypted way which is immutable and isolated from other IT areas of the company, through the usage of Distributed Ledger Technology (DLT) solutions. Such automation accounts for the transparency principle. Obviously, its contribution must be understood as not being attached to the any particular DLT ledgers, but as a convenient data management approach to show its capabilities.
- Validation of the proposal through Proof of Concept, as deployed in an industrial case involving a real manufacturing company.

Therefore, in Section 2, we review some background principles and contributions related to attributes connected with the value proposal. Section 3 introduces the proposed architecture, including the reference updated framework and its significant components. Section 4 presents the scenario for proof of concept, involving several devices with different semantic meanings in such a way that their data flows are integrated. In Section 5, a discussion of the business dimension is carried out. Finally, the last section is devoted to presenting our conclusions.

## 2. Literature review

There are several principles that need to be considered when a new or improved framework is presented. These principles are:

- *Data silos*. At present, data produced by IIoT deployment devices and wearable devices are controlled or owned by different device manufacturers. Most of the existing value proposals for the IIoT deployment or wearable devices include one platform to manage the physical devices in an integrated way, as well as collecting the data from them by placing it into a private cloud for processing [32,33]. Benefiting from Radio-frequency identification (RFID) and sensor network technologies, common physical objects can be connected and are able to be monitored and managed by a single system [34]. However, as increasing types of IIoT and wearable devices from different vendors become available, more data silos are generated. The existence of such data silos not only jeopardize potential data-driven services and applications; the limited and restricted features provided also dramatically hinder the learning and inference capability derived from the higher requirements of data integration. Most importantly, all related stakeholders in I4.0 are looking forward to a transparent and shared information platform including production and operator data, which is difficult to realize due to the security constraints when passing between various data silos.
- *Data ownership*. It is an additional challenge for data producers (normally referred to as operators) to reuse (e.g., monetize) their data outside the data provider’s environments. The data producer cannot benefit from the data they generate, as the data is locked inside of the internal data environment of the enterprise. They lose their data ownership and the business opportunities stemming from those data are outside of the data owner’s control. Under this perspective, when referring to data related to humans, the EU General Data Protection Regulation (GDPR) is as effective tool to add significant trust to this dimension, as users are now able to understand their rights and privacy.
- *Privacy*. When data are related to people, such as devices which are linked to apps by bluetooth, the apps are mostly designed to present the data to users and upload summaries to the cloud manufacturer [35]. However, data integration (especially when related to people) has limitations related to privacy and misuse [36,37]. Cyber security is vulnerable, as wearable device manufacturers have reduced their safety protocols and safety stack layers to enable cheap products, as they have been understood as only serving the end user in a local context. Therefore, there is a clear demand regarding the concept of building and embedding security and privacy controls into connected products, as well as the infrastructure itself. This is one of the implementations of the Privacy by Design (PbD) concept [38,39].
- *Interoperability*. IIoT devices, including wearable devices, are highly heterogeneous in terms of the underlying communication protocols, data formats, and technologies from different vendors. Such heterogeneous infrastructures, devices, and configurations have becomes a strong limitation for data integration and interoperability. By 2021, 25 billion sensor-enabled objects are expected to be connected to the IIoT, as reported by Gartner [40].

Traditional factory-floor control and interconnection data management solutions are mostly based on centralized systems. The German Federal Ministry of Economic Affairs and Energy has provided specifications for the exchange of information within the administration shell. The Open Platform Communications Unified Architecture (OPC UA) [41] is the core communication standard for I4.0-compliant communications [42]. Its adoption was a significant step towards the interconnection of devices, according to RAMI 4.0.

RAMI 4.0 is based on a three-dimensional co-ordinate system consisting of the Layers, Life-Cycle and Value Stream, and Hierarchy Levels dimensions, according to the IEC 62890 and IEC 62264/IEC 51512. It is derived from the Smart Grid Architecture Model (SGAM), which is a key outcome of the EU Mandate M/490 Reference Architecture group for the purposes of communication in networks of renewable energy sources.

In addition, several other proposals aimed at contributing to such common perspectives among stakeholders have been proposed by different institutions. Therefore, The National Institute of Standards and Technology (NIST) has promoted the Smart Manufacturing vision as collaborative manufacturing systems which are able to cope with the challenges of quality, efficiency, and personalization that manufacturing companies are presently facing. China has proposed the National Intelligent Manufacturing System Architecture (IMSA), looking to guide the upgrade of Chinese manufacturing towards intelligent manufacturing. However, wearable devices have been neither well-considered to be connected elements in the Integration Layer of RAMI4.0 [13], nor in the 5C architecture [12].

After RAMI 4.0, the Industrial Internet Reference Architecture (IIRA) for IIoT Systems has been introduced, promoting IIoT architects to use a standard-based architecture framework and adopt a common vocabulary based on ISO 42010. All of these frameworks have been discussed in [42], although such architectures did not specifically include the operator’s contributions.

Compared with RAMI 4.0, the LASFA model can not only enable more reliable and simple modeling of smart factories, but also cover the functionality of Enterprise Resource Planning (ERP), Manufacturing Execution System (MES), and Product Lifecycle Management (PLM) sub-systems with new digital twins, which are integrated digital agents supported by artificial intelligence. Approaches such as cloud-based data storage methods have created the centralized approach, shifting the way in which franchisers interact with franchisees [32,33]. However, such systems (e.g., Programmable Logic Controllers (PLCs)) are expected to be too limited in their accessibility to keep up with the growing demand for higher level integration [43] and information transparency for all relevant stakeholders [44,45].

Distributed ledger technologies (DLTs), such as blockchain, provide a convenient replacement for the central administrator in guaranteeing the integrity of a database. Blockchain-based data sharing systems [46] were proposed to address data security and privacy issues and have gained trust from data owners [47,48]. However, blockchain-based protocols still have several drawbacks that hinder their usage in generic data IoT data sharing platforms [49]. The inherent transaction rate limit leads to low throughput, which brings about scalability issues. The concept of a transaction fee for any value transaction is difficult to get rid of in blockchain-based IIoT infrastructures and some concerns have been raised regarding the susceptibility of blockchains to quantum computers [49].

The lack of interconnected data and shared meaning is another limitation for most DLT-based IIoT data platforms [50,51]. Most sensor data sources, including wearable devices, are characterized by a high degree of heterogeneity, and the implementation of their provided interfaces is highly dependent on the underlying device hardware [52]. These data silos with heterogeneous data jeopardize potential data-driven services and applications. Delicato et al. [53] pointed out that discovering and retrieving data from IoT is a challenge, which is worsened by a lack of standardization of formats in representing resources. However, efforts are still slow-paced to create interoperable platforms to bridge such huge data silos.

To ensure data privacy and to protect data ownership, the data need to be digitally signed and encrypted; even in a distributed platform supported by DLT. Besides blockchain-structured protocols, some recent DLTs have been developed which are specifically designed for the IoT industry. For example, the Directed Acyclic Graph (DAG)-structured IOTA surpassed conventional blockchains, in terms of its scalability, fee-less, and quantum-resistant features [54]. In the IOTA network (named the Tangle), a new transaction needs to approve two previous transactions to attach itself to the network; this transaction will then be validated by some subsequent transactions [49,54]. This mechanism theoretically eliminates throughput caps as the more transactions that are added, the faster the approval speed is.

IIoT requires interconnected data for predictive maintenance, industrial automation, operational efficiency, and better decision-making. Extending the ability to use sensor data integrated from a wide variety of heterogeneous sources facilitates the building of multiple applications and services, which is a vital step toward the success of the IIoT vision and I4.0. However, to effectively address the interconnection of data from heterogeneous entities ranging from simple sensing devices to complex robotic devices, service agents, or human actors in a consistent way, it is necessary to pay attention to the meanings of the individual data streams [55]. The functions of the information layer in LASFA+ also demand consistent integration of higher-quality data, with the provisioning of structured data [56].

Aimed at the data interconnection issue, semantic modeling (e.g., ontology-based techniques) proved successful in dealing with data interoperability and semantic compatibility [57,58] in the manufacturing domain. The Blockchain ontology with dynamic extensibility (BLONDiE) ontology [59] has been developed for Bitcoin and Ethereum but is still in its initial phase. Upper level ontologies, such as the Basic Formal Ontology (BFO) [60] and Descriptive Ontology for Linguistic and Cognitive Engineering (DOLCE) [61], work as hub-and-spokes strategies for bringing about interoperability throughout a domain. Ontologies, thus, are in development in various areas. BFO is currently being used in industry IT contexts to gain interoperability in digital manufacturing. Based on BFO, the aim of the Industry Ontology Foundry (IOF) [62] initiative is to create a suite of interoperable and high-quality ontologies covering the domain of industrial (especially manufacturing) engineering [63].

Integration not only happens among the data streams of wearable devices, but higher levels of knowledge can also be derived in industrial contexts when integration between production processes and operator data is considered. This allows more integral access to the infrastructures by means of digital twins [64].

To overcome the aforementioned concerns, this study improves upon an interoperable data handling architecture. The semantic modeling module encompasses heterogeneous resource handling by applying an ontology-based information model, where the ontologies take advantage of well-established and standardized concepts and relationships derived from existing ontologies, in order to semantically represent data resources in IIoT [65].

## 3. Proposed Architecture

To complement the RAMI 4.0 reference model, and taking advantage of the LASFA architectural model, we integrate all the key elements and their interconnections in a two-dimensional platform. It includes several production layers, as well as a business process. The production layers represent the manufacturing company in terms of production lines, production cells, warehouses, and manual workplaces, creating the local production systems (which are treated as distributed systems). The business layer of the LASFA model includes the company’s strategy, business planning, and the monitoring and the delivery of orders [14].

As indicated in Section 1, our main ambition is to extend the proposal of the existing, modern, and valuable framework, LASFA. The general structure of the LASFA architectural model was presented in [14], and its components and attributes were compared with those of RAMI 4.0 in Section 4 of the above reference.

It is evident that LASFA is much more specific and offers the end-user a simple visualization of the entire architecture of the smart factory, with the definitions of the exact locations and functions of the digital twins and agents, as well as the specific information and data flows between them. The model shows, very clearly, the distribution and autonomy of every single building block in the smart factory, from the product to the management.

**Figure 2:**
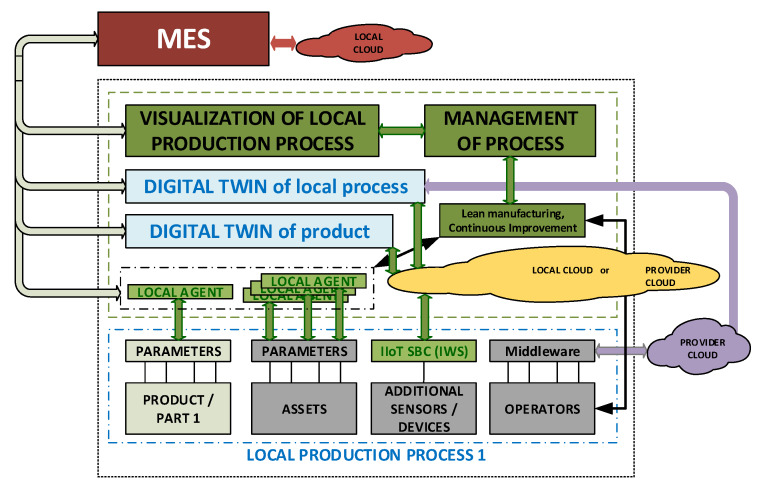
Improved lower layer for the LASFA+ architectural model. MES: Manufacturing Execution System.

In general terms, the LASFA architecture fits with the expectations raised in this work. However, it does have several limitations and, so, this paper adds some extensions to create the improved LASFA model, which we call LASFA+ (see Figure 2).

The first difference is the wider perspective given to the elements participating in production, where not just traditional assets are considered but also extended ones, providing complementary information to the production process. Some of those services rely either on local or (more frequently) on provider-based clouds outside of the company boundaries. Therefore, the reference model needs to be consistent with such realistic conditions. Obviously, such data flows should be integrated with the traditional ones to build the digital twin.

Another difference is the role of the MES and ERP systems, where the MES used to be closer to production and as a support for visualization of the data of the production process.

Machines, devices, and people are equipped with sensors in order to connect and communicate with each other. This proposed architecture enables the connection of people by low-cost wearable devices which can be conveniently obtained in the market, such as personal health-tracking devices. This architecture makes it possible to not only collect physical features and events but also virtual ones, as a result of the model’s output. This becomes useful, when integrated, to develop even more complex models.

The information from various sensors can be further processed in edge devices, such as local servers and single-board computers, where such processing includes data fusion, semantic modeling, and data encryption.

Data fusion is relevant to different aspects, and can include data summaries over certain periods of time from a certain type of sensor. To provide a higher view of data, raw data may need to be statistically summarized. Extracting data patterns or classification of raw data to high-level knowledge could also be included in data fusion. Machine learning (ML), deep learning (DL), or inference mechanisms can be applied for this purpose. A local server is preferable in this context, considering the high computation demands of ML or DL methods. The motion/positioning data collected from sensors can be segmented and classified into different types of activity, such as running, walking, and so on.

To enable data interoperability, semantic information is annotated in each data message. Existing ontologies in any domain (besides industrial) are able to be reused under this architecture. Ontology-based structured data can be conveniently integrated with other data sets, in order to empower more complex applications (e.g., data-driven AI algorithms). Ontologies aligned with existing ones or newly proposed ontologies are also supportive in this semantic modeling module.

**Figure 3:**
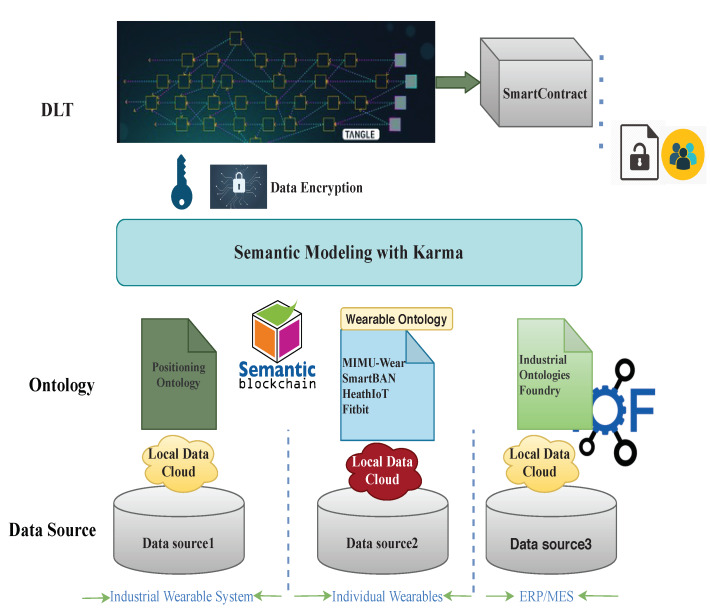
Prototype of data handling in an industrial context. DLT: Distributed Ledger Technology; ERP: Enterprise Resource Planning; MES: Manufacturing Execution System; MIMU: Magnetic and Inertial Measurement Units.

As shown in Figure 3, an application scenario was developed which enables interoperability among different data sources, further providing a secure, distributed database that empowers data access by different stakeholders. The main components of the prototype include a heterogeneous data source, ontology-based interoperability modeling, and incorporation within the DLT module for data dissemination.

The corresponding ontology schema is functional as an information model for the automatic understanding of data during retrieval procedures. Data on the DLT, such as the IOTA Tangle, can be retrieved by data consumers. Various data transactions can be retrieved by having the address/tag/bundle or integrated based on defined ontology schema. In this way, the dominant paradigm of keeping data hidden from public knowledge by having protected access is broken and the data becomes part of the product being delivered for further usage. Data consumers may define their own ontology fitting their specific requirements, where this data will be seamlessly integrated.

Although data published to a DLT (e.g., IOTA Tangle), is protected in a secure infrastructure, the message within each transaction confronts a data security issue. If the message is not encrypted, once the transaction address is spread in the web, any party who has the tag, address of the receiver, or transaction ID is able to read the contents.

Here, different policies can be adopted, such as:

- *Public.* This is the already-described mechanism, where anyone knowing the address of the receiver has the right to freely access to the content.
- *Restricted.* This is the case where sensitive information needs to be shared among only a limited number of stakeholders. To prevent unauthorized entities from reading the data, the message content is obfuscated by encrypting it before uploading it to the Tangle. DLT access at message level, including the encryption/decryption process, is demonstrated in Figure 3.
- *Private.* In this case, due to the specific content of the data, to prevent unauthorized entities from reading the data and to respect the GDPR enforcement rules, the message content is obfuscated by encrypting it before uploading it to the Tangle by using a private certificate, making it Private by Design (PbD).

For messages which are determined to be *Private*, the framework proposed in this paper can be extended in such a way that data owners are able to share a concrete data set defined by matching some conditions (e.g., a period of time) with specific entities. The proposed solution is that the data owner installs a web-service which is able to handle such authorized data requests. The signature key of the data structure will be a public one, preserved under a smart contract and consumed by a web service which is in charge of providing the requested information. The designed data structure for the request can be provided in JSON format for the REST-type implemented protocol, which includes the following elements,

- The DLT address, representing the interested entity or device;
- The DLT tag, representing the interested data set;
- The public key, for the user requesting the access to the DLT stored data;
- Selection criteria for the required data, such as from [DateTime] to [DateTime]; and
- Proof of worth for accessing the data.

When either people or agents request the web service to access the specific data according to the above structure, when they have the right of access, the web-service will access the DLT repository under the specific criteria provided and verify the ownership of the requested information. This verification will be based on consuming the smart-contract to obtain access to the symmetric encryption key used by the node, thus being able to decrypt it and compare signatures. Then, such data can be aggregated (according to the requested period of time) and be sent to the requester by the web service after encrypting it using the provided public key of the user/agent. The data consumers will receive back the data and handle it by using its private key, in such a way that only they themselves are allowed to consume it.

**Figure 4:**
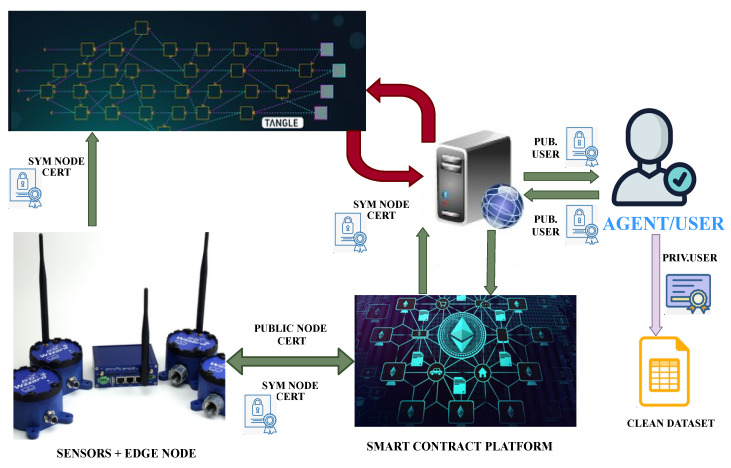
Global schema for Web Services operation including certificate usage.

The whole schema can be seen in Figure 4, where the role of different certificates is shown clearly. In this way, no third parties are aware of the encrypting/decrypting certificate, as the data is made available to them by re-encrypting it with their public asymmetric certificates, which only enables the third party to decrypt its content.

This type of solution can help companies from at least three different perspectives. They can redesign their adopted business models related to the IIoT and its integration into the management dimension of the organization [66,67]. However, it may also become useful to increase the knowledge of the existing processes in the existing business model, or may just help in the update process of such business models themselves.

In the case of public messages, there are no requirements for encryption. Under the restricted approach, the encryption certificate can be shared among the interested stakeholders, in such a way they can collect the information on their own.

Not only due to the transparency provided, but also because such data streams are delivered automatically by the different digital twins, the trust of shareholders and consumers will increase immediately. In addition, such knowledge will enable the management needs of owners, being more consistent with its potential value when shared.

## 4. Proof of Concept: An Industrial Scenario

To validate the proposed architecture, a data-handling system based on a DLT (IOTA), and domain ontologies was designed to improve data interoperability, with integrated privacy criteria through message encryption and data sharing under the Industry 4.0 paradigm.

The implemented system was adopted in a practical industrial scenario. The aim was to verify that our proposed architecture has practical potential in real-world applications, leveraging the implemented system to demonstrate its feasibility. The approach, which adopted a positivist view of research, relied on the literature and empirical data coming from the case itself, as well as on the insights of the researcher to build incrementally more powerful theories.

**Figure 5:**
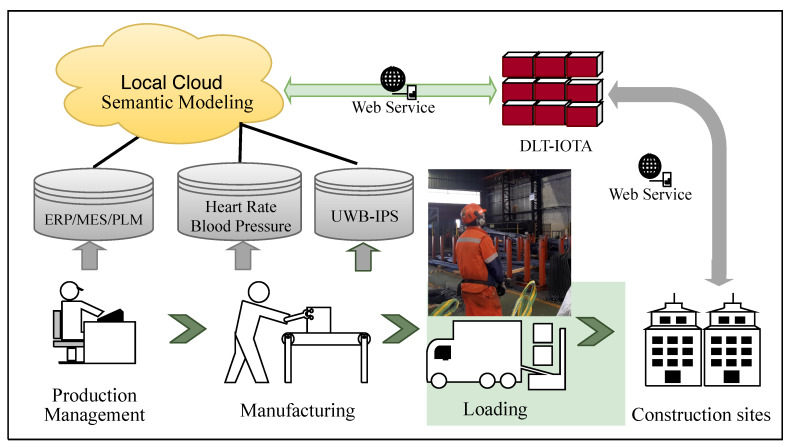
Application scenario of the proof of concept implementation. DLT: Distributed Ledger Technology; ERP: Enterprise Resource Planning; MES: Manufacturing Execution System; PLM: Product Lifecycle Management; UWB-IPS: Ultra Wide Band Indoor Positioning System.

The application case was conducted in a Spanish factory manufacturing steel rebars to reinforce concrete in the construction sector. The general workflow is as shown in Figure 5. Its production process (operation) includes cutting, blending, and welding rebars into different size configurations, packing them into rebar bundles according to customer’s order, and loading the rebar bundles into trucks for logistics. For each process, there are different data flows that may affect the production efficiency and effectiveness; the data flows came from data sources such as production line, IIoT divides such as industrial wearable systems (IWSs), and personal wearable devices.

Consider the truck loading process as an example. The requirement is that the right rebar bundles are loaded into the truck in a specific disposal sequence, which needs to be well managed as the bundles are delivered and distributed to different sites. As a lack of specific items could impact the delivery dates, an improper loading disposal sequence may negatively affect the scheduled work in the unloading process. In the present situation, the responsibility for such a decision relies on the crane operators, who take charge of loading the rebar bundles to the truck buffer using a crane machine.

To effectively assess this internal logistics, different data sources should be taken into consideration. Such an analysis requires knowledge about the order and manufacturing sequences, which are handled by the ERP system, MES, or PLM. The logistics information is required, such as where and when the items are loaded into the truck, in order to understand whether the items were loaded and well-placed in a proper storage buffer in the truck.

Moreover, more details, such as the crane operator profile, their working conditions, and working status (e.g., health parameters and movement trajectory/speed) are also relevant. Body-related parameters need to be integrated, mainly as the stress of the crane operators may affect the loading process. The movement trajectory/speed need to be considered, as analysis can be made to understand whether the loading process requires excessive physical efforts.

When different stakeholders need to have access to specific process-related information, non-tampered data are required due to certification principles; therefore, an open system is a convenient tool, reducing the IT barriers and which is robust against facility ownership changes.

The system prototype will adapt the proposal presented in Section 3 by adopting specific mappings over the particular characteristics of the company and its processes.

Different devices, systems, and data sources composed the configured prototype, as indicated below:

- Production related information from ERP/MES/PLM system;
- Ultra wide band (UWB) indoor positioning system (IPS) to track crane position and crane operator movements, to better define the location for rebar bundles; and
- Smart band to monitor crane operator’s heart rate and blood pressure.

The MES manages sequence planning and the bottom-up data flow on the shop-floor [68]. It provides the current state of the process, identifying the optimal resources allocation by taking consideration of the workstation capacity and organization-level requirements (e.g., manufacturing order). All data generated by manufacturing processes are stored in a single database in the company local cloud, based on a Microsoft SQL server database.

The UWB indoor positioning system (IPS) from the Tracktio™ company (https://tracktio.com/) was used to track the crane hook and crane operators. Rebar bundle locations in different buffer areas of production or positions in the truck for customer delivery were derived from the dynamic behavior of the process. The position of crane operator was tracked as a reference for understanding the movement trajectory and speed. The IPS had its own data repository; the data were acquired from a web service.

A smart band was worn by the crane operator, in order to monitor their stress factors, such as blood pressure and heart rate. Every minute, data was collected and uploaded to a smart phone (as an example of edge computing); then, it was reprocessed and transmitted to a MongoDB database located in the local cloud.

For data interoperability, all data sources in this industrial scenario were semantically modeled, fostering their linkage to other domain knowledge. To this end, different existing domain ontologies were reused in this study. The data sources were in the three aforementioned sectors: production system, IPS, and individual wearable devices. The existing and shared/published ontologies for each sector were collected and one was selected to model the data source in this study, according to the mapping of data structure. The details of available ontologies for reusability are listed in Table 1.

To map the generated data sources in this industrial scenario, three ontologies were chosen for data modeling. The selection criteria were chosen on basis of the degree of matching between the ontological schema and data source structure. After deeper analysis of each ontology’s schema, as listed in Table 1, the vital sign ontology [85] was used to model heart rate and blood pressure data from the Smart band; the rebar bundle and crane operator’s position were modeled with the positioning ontology [69]; and the MES ontology [72] was applied for the model production system data source.

The mapping tool Karma (https://usc-isi-i2.github.io/karma/) provides a graphical user interface which automates the semantic modeling process. Karma learns to recognize the mapping of data to the chosen ontology classes and proposes a model that can generate JavaScript Object Notation for Linked Data (JSON-LD) for large data sets in a batch mode. JSON-LD is a lightweight Linked Data format, which was designed based on the concept of "context", linking object properties in JSON to concepts in an ontology. The JSON-LD data type was selected as it is lightweight and interoperate at Web-scale, but also provides embedded semantics. The data format is based on JSON and ontological schemas, maintaining a common space of understanding and supporting the evolution of schemes over time without requiring data consumers to change format. The data source, applied ontology schema, and the transformed JSON-LD format (containing semantic annotation) are listed in Table 2.

To distribute information about the process interaction, the IOTA network Tangle is required. For this, access to a node is needed and a public node (https://nodes.thetangle.org:443) was selected for data submission to the IOTA Tangle. The message sent to the IOTA Tangle is encrypted. A python script that implements the RSA (Rivest–Shamir–Adleman) public-key cryptosystem was used for encryption and validation of messages. PyOTA (https://github.com/iotaledger/iota.py), an IOTA python API library, was used to implement data sending and retrieval to and from the IOTA Tangle. The source code can be found at [86], which supports Python and NodeJS.

A data message describes relevant information combined from different data sources, in which the details about loading a rebar bundle are encrypted and delivered to the IOTA Tangle by the IIoT system, after integrating the meaningful information. In this application, every bundle loaded into a truck and delivered to a construction site was encoded as a message. As the unit is the truck, every truck has a specific encryption certificate, which could be shared with interested stakeholders (e.g., construction workers, transport agencies, insurance companies, or end consumers).

**Figure 6:**
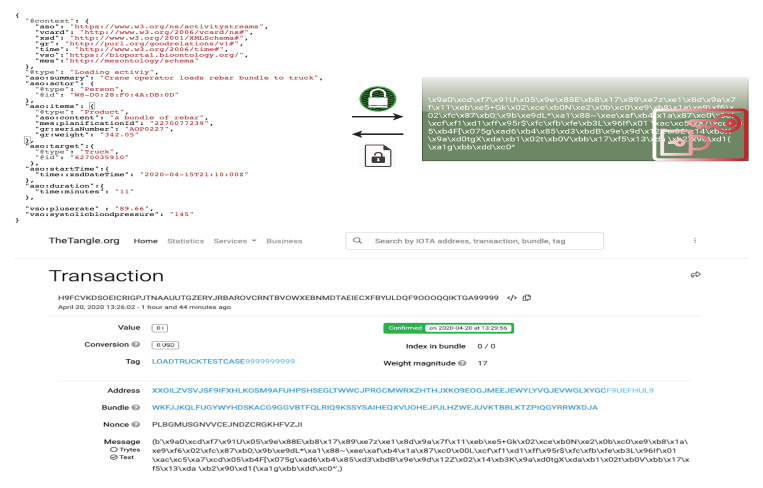
Data handling process supported by IOTA Tangle; loading activity as example.

As shown in Figure 6, the loading truck activity is labeled with the tag *[LOADTRUCKTESTCASE]* in IOTA Tangle. Related transactions could be looked up and fetched by tags or bundles. After data retrieval, a data message should be validated and decrypted by stakeholders, where the semantic meaning of the data are encoded in the message itself. This design fosters data reusability by learning processes and growth of models, as they can select the appropriate meaning of data sets or ask for automatic preprocessing before accomplishing further transformations.

## 5. Discussion

The needs of the near-future dimensions of I4.0 require an increasing level of integration of data from different sources, including data from not only workers, but also from different providers and sources. This enforces PbD requirements, due to both company security rules and regulatory requirements. A major aspect of the GDPR are the so-called legal grounds for lawfully processing personal data, one of which is consent. In several IoT applications where consent is used, it may even need to be explicit consent.

There still remain, after the GDPR, some problems with personal data, as follows:

- Data consistency and the use of different sources of information and/or different time periods or geographical positions;
- Data storage: although properly scrambled, masked, or blurred, the related persons probably are not aware such pieces of information do exist related to them; and
- Problems related to EU citizens when requesting products or services outside of the EU.

The harder problem is the second one, mainly due to the absence of accountability: as a user never knows who is gathering information about them, they cannot ask for access, erasure, or modification. Thus, there are two types of accountability:

- Accountability regarding users; but also,
- Accountability within the organization.

In our validation case, the delivery process to the customer has limited information from the process itself, as truck loading is mainly a human-driven activity. Therefore, it risks exposure to a significant time variability and existence of errors in missed or items wrongly included inside the truck. From a business perspective, adding transparency by maximization in the amount of automation involved is worthwhile for stakeholders. Furthermore, current private and centralized data management approaches limit the high-level integration of multi-modal data sources, as well as data ownership and re-usability (e.g., monetizing health data generated from data producers).

The deployed architecture, as proof of concept, was able to provide additional information, integrated in a convenient way in order to help to understand the type of items frequently creating problems, configurations of truck layout consistent with specific delivery sequences, the movements used to load particular item shapes, and so on.

There exist potential contributions in the direction of empowering operators of I4.0 [87], including virtual and augmented reality applications to facilitate the operator’s activities and to reduce unwanted mistakes or damage by giving appropriate instructions for complex tasks or working environments. They are also useful for job role allocation and/or for determining training needs, especially in dynamic work environments with changing conditions [88]. Therefore, the impact of the physiological conditions of workers can be considered to be relevant, especially the influences of tiredness or stress.

Obviously, to have significance in such an analysis, large periods of data collection and close integration are required. Indeed, path dependency [89] does exist; however, such analysis exceeds the scope of the present research.

Another critical enabling technology for data interoperability adopted in this study is semantic modelling and ontology engineering. Ontologies provide standardized definitions for different data sources and make possible of reasoning and autonomous decision-making, by formalizing the structure of the knowledge. Existing ontologies, i.e., vital sign ontology, positioning ontology, and the MES ontology, are reused in this study to accelerate the development. To obtain a higher level of interoperability, such ontologies can be further exploited and adapted to keep align with widely recognized upper-level ontology such as BFO [60] and to be attached to industrial ontology initiatives such as IOF [62]. In this way, the developed ontologies will be able to interact with other domain ontologies thus to achieve wider range of interoperability in I4.0 context.

As one of the fundamental enabling technologies of I4.0, the digital twin concept is briefly discussed in this study but not explored with details as it is not the main focus of this paper. Nevertheless, the proposed data integration and handling approaches are important basis for implementation of digital twins. For example, in some existing digital twin studies [90,91], similar semantic modelling techniques have been adopted to improve data interoperability data heterogeneous issues. Aiming to identify the dynamics of virtual model evolution and enhancing the decision-making capabilities, the next generation of digital twin, i.e., cognitive twin concept, has been proposed in a recent study [92], which integrated augmented semantic capabilities with conventional digital twins. The work presented in this paper will have great potential in these digital twin and cognitive twin domains.

## 6. Conclusions

In this paper, our main contributions were to extend and complement the I4.0 reference model LASFA, with reference to some more general ones (e.g., RAMI4.0), by adding additional data flows to complete the production process description and including different provider add-ons for digital twin construction. The proposed update enables higher level integration and more efficient process description. By considering the PbD design for exchange of information over public DLT systems, it also accounts for information transparency and data ownership for related stakeholders.

The adopted configuration, which enables the semantic enrichment of data, also make it possible to implement rules enforcing interaction mechanisms and to derive new properties related to the employed ontologies. Although such a direction was not implemented in the proof of concept carried out, it may be useful for stakeholders accessing the data.

The presented proposal benefits from the integration of semantically annotated data from different sources, including human health-related wearable device data, in an industrial context. When information related to people has been included, a signature layer is used to provide a PbD solution, in full accordance with the GDPR regulations and to enable positive-Sum [39].

The core idea of the proposed architecture is to enable the sharing of different obfuscated data streams over a public immutable network-oriented database with quick-answer capabilities and good scalability characteristics. The proposed architecture enables extensions, in order to facilitate higher levels of control and empowerment of data owners by integrating an explicit delegation of access to specific data sets and time windows through the use of web services.

Considering a simple application case, the benefits from a business perspective appear evident, with the integration of different dimensions not previously considered. There also exists additional value with the possibility of further performance analysis of procedures, when the proposed architecture is combined with artificial intelligence (AI) techniques. The theoretical implications of this research allow for a convenient framework providing a high level of control on the data produced by each agent, while also providing a platform to share data coherently with different business models. It also provides a flexible way to interconnect semantically powered data streams, which is a significant contribution for data driven ML/AI applications.

This paper also makes a point from a practical application perspective; in particular, by providing an interesting implementation of data handling strategies aligned with Industry 4.0 principles enabling the integration of data coming from workers. Such tools can have a significant impact on advanced lean manufacturing and lean management implementations, as they go a step beyond classical digitization approaches. This could be the case for the standardization of formal communications under Lean Management (CPD)${}_{n}$A.

There are several aspects that provide us with further research paths, as we require long data collection approaches and the definition of consistent key performance indicators for individual applications. Furthermore, the definition of related business models for micro (smart contract-based) data monetization and traceability applications also provides avenues for further research.

A clear limitation of the existing research is the adoption of a single and specific DLT technology; namely IOTA. The proposal is not conceptually dependent on IOTA; it simply served as a driver to implement our architecture in the proof of concept. However, some other technologies, such as Obyte, may be considered to be potential substitutes.

Another limitation is the required digital infrastructure needed in the companies to successfully implement the digital twin concepts, which is capable of managing requirement of both internal and external stakeholders.

Future research steps will be to deeply analyze the meaning of the integrated data built, in terms of value for business, looking to identify behavioral patterns or to create forecasting models, and its ability to better adjust operation times and, most importantly, timely delivery.

Another interesting perspective to be considered is that wearable devices, such as smart watches [88] and smart wristbands, can be useful for estimating the operator’s well-being (i.e., cognitive, psychological, and physical needs) in Human Cyber–Physical Systems (H-CPS) [93]. Wearable monitoring provides opportunities for health diagnostics and operator well-being in industrial environments, as was also identified in [94,95].

## Figures and Tables

**Table 1:** Ontology Reusability Selection.

Ontology List		
**IPS**	**Wearable**	**Production System**
Positioning Ontology [69,70]	MIMU-Wear Ontology [71]	MES ontology [72,73,74]
IndoorGML [75]	SmartBAN Ontology [76]	ERP ontology [77,78]
Navigation ontology [70,79,80]	HealthIoT Ontology [81]	PLM ontology [73]
Indoor space ontology [82,83]	Fitbit Ontology [84]	
	Vital Sign Ontology [85]	

ERP: Enterprise Resource Planning; IPS: Indoor Positioning System; MES: Manufacturing Execution System; MIMU: Magnetic and Inertial Measurement Units; PLM: Product Lifecycle Management.

**Table 2:** Data semantic transformation based on ontology and Karma.

Semantic Modeling			
**Data Source**	**Data Storage**	**Ontology**	**JSON-LD**
MES (ISA-95)	Microsoft SQL database	MES ontology [72]	{ "@context": { "mes":"http://mesontology/schema", "gr": "http://purl.org/goodrelations/v1#", "pto": "http://www.productontology.org/id/", "xsd": "http://www.w3.org/2001/XMLSchema#", "gr:seriaNumber": { "@type": "xsd:int" }, "gr:description": { "@type": "xsd: string" }, "gr:amountOfThisGood": { "@type": "xsd:int" }, "gr:eligibleRegions": { "@type": "xsd: string" }, "gr:weight": { "@type": "xsd:float" } "mes:planificationId": "2270077239", "gr:seriaNumber": "AAAEEE3452XX", "gr:description": "bundle of rebars", "gr:amountOfThisGood": "20", "gr:eligibleRegions": "DE(Germany)", "gr:weight": "300", "gr:includes": { "@type": [ "gr:Individual", "pto:Rebar" ]} }
Smart band	MongoDB	Vital Sign Ontology [85]	{ "@context": { "vso": "https://bioportal.bioontology.org/ ontologies/VSO", "xsd": "http://www.w3.org/2001/XMLSchema#", "vso: pulserate": { "@type": "xsd:int" }, "vso: systolicbloodpressure": { "@type": "xsd:int" }, "sumo": "http://www.adampease.org/OP/SUMO.owl", "sumo:timepoint": { "@type": "xsd:dateTime" }, "vso:pulserate" : "79", "vso:systolicbloodpressure": "111", "sumo:timepoint": "2020-04-08T11:20:00Z" }
IPS (tracktio)	CSV file	Positioning Ontology [69]	{ "@context": { "positionpoint": "http://positioningontology/schema", "xsd": "http://www.w3.org/2001/XMLSchema#", "axis": "http://data.ign.fr/def/ignf#CoordinateSystemAxis", "axis: listofaxes": { "@type": "xsd:list" }, "sumo": "http://www.adampease.org/OP/SUMO.owl", "sumo:timepoint": { "@type": "xsd:dateTime" }, "positionpoint": { "axis: listofaxes": "7.299998 1.140002 1.499998" }, "sumo:timepoint": "2020-04-09T10:00:00Z" } }

IPS: Indoor Positioning System; JSON-LD: JavaScript Object Notation for Linked Data; MES: Manufacturing Execution System.

## Abbreviations

The following abbreviations are used in this manuscript:

AI	Artificial Intelligence
DAG	Directed Acyclic Graph
DL	Deep Learning
DLT	Distributed Ledger Technology
ERP	Enterprise Resource Planning
GDPR	General Data Protection Regulation
I4.0	Industry 4.0 paradigm
IIoT	Industrial Internet of Things
IoT	Internet of Things
IPS	Indoor Positioning System
IT	Information Technology
IWS	Industrial Wearable System
JSON	JavaScript Object Notation
JSON-LD	JavaScript Object Notation for Linked Data
KPI	Key Performance Indicator
LASFA	Lasim Smart Factory reference model
MES	Manufacturing Execution System
ML	Machine Learning Techniques
OPC UA	Open Platform Communications Unified Architecture
PbD	Privacy by Design
PLM	Product Lifecycle Management
UWB	Ultra Wide Band
